# Electromagnetic surface waves supported by a resistive metasurface-covered metamaterial structure

**DOI:** 10.1038/s41598-020-72396-7

**Published:** 2020-09-23

**Authors:** M. Z. Yaqoob, A. Ghaffar, Majeed A. S. Alkanhal, M. Y. Naz, Ali H. Alqahtani, Y. Khan

**Affiliations:** 1 Department of Physics, University of Agriculture, Faisalabad, Pakistan; 2 Department of Physics, Government College University, Faisalabad, Pakistan; 3 Department of Electrical Engineering, King Saud University, Riyadh, Saudi Arabia; 4 Department of Electrical Engineering, College of Applied Engineering, Al-Muzahimiyah Branch, King Saud University, Al-Muzahmiya, Saudi Arabia

**Keywords:** Engineering, Optics and photonics, Physics

## Abstract

This study examines the analytical and numerical solution of electromagnetic surface waves supported by a resistive metasurface-covered grounded metamaterial structure. To simulate the metamaterial, the Kramers–Kronig relation based on the causality principle is used, while the modeling of the resistive metasurface has been done by implementing the impedance boundary conditions. The analytical expressions for the field phasors of surface waves are developed for the transverse magnetic (TM) polarized mode and transverse electric (TE) polarized mode. The characteristic equations are computed for both modes, and the unknown propagation constant is evaluated numerically in the kernel. After computation, the dispersion curves, electric field profiles, effective mode index ($N_{eff}$), and phase speeds ($v_{p}$) are presented for both the TM and TE polarized modes. To study the tunability of surface waves, the influence of the thickness of the metamaterial slab ($d$), effective permittivity of the metamaterial ($\varepsilon_{1}$), thickness of the resistive metasurface ($t$), and effective permittivity of the metasurface ($\varepsilon_{r}$) on all the numerical results has been studied. However, the geometrical parameters are found to be more sensitive to the effective mode index ($N_{eff}$) and phase speed ($v_{p}$) of the surface waves. The results are consistent with the published results, which reflects the accuracy of the work. It is concluded that the appropriate choice of parameters can be used to achieve surface waves with the desired characteristics in the GHz range. The present work may have potential applications in surface waveguide design, surface wave speed controllers, surface communication devices, and light trapping configurations.

## Introduction

Two-dimensional metamaterials of subwavelength thickness with the inherent ability to control, modulate, and manipulate electromagnetic energy are known as metasurfaces or metafilms^1^. Metasurfaces have groundbreaking applications, such as wave shaping, phase controlling, focusing, imaging, surface cloaking, surface waveguides, leaky wave antennae, twist polarizers and deflectors^2–7^. Generally, the designing of metasurfaces is being done by adding the two-dimensional inclusions at the subwavelength level or by printing the metasurface unit cells on the desired surfaces that can manipulate light at the subwavelength scale^8^. To synthesize planar unit cells or patterns, conventional surface lithography techniques (e.g., photolithography, electron beam lithography, focused ion beam lithography) are being employed, as given in^9^, which enhance the practical applicability and realization of such metasurfaces. Impedance metasurfaces, resistive metasurfaces, selective frequency metasurfaces, metasurface lenses, Huygens’ surfaces, nonlinear metasurfaces, non-reciprocal metasurfaces, bi-anisotropic metasurfaces, and graded index metasurfaces are some typical types of metasurfaces^3,10–12^.

Recent developments in surface optics allow us to control and manipulate the electromagnetic energy on the surface for the sake of communication, modulation, sensing. Many schemes have been proposed and studied for the generation of surface waves along the interface of dissimilar media^13,14^. For example, (1 the metal–dielectric interface supports surface plasmon polariton (SPP) waves, (2) the isotropic dielectric-anisotropic dielectric interface supports Dyakonov surface waves, and (3) Zenneck waves propagate along the interface between two dissimilar dielectric media^15^. To tune and manipulate these surface waves, many active materials and schemes have been proposed^16,17^. To achieve extraordinary control over the surface waves, Cory and Barger studied surface wave propagation along a metamaterial slab with simultaneous negative permittivity and permeability. The dispersion relations and cutoff frequency range for both the transverse magnetic (TM) and transverse electric (TE) modes were determined, and the propagation of surface waves was reported for both modes^18^.

Baccarelli et al. extended the work and reported the modal solution of a grounded metamaterial slab for all configurations of metamaterials [i.e., double positive (DPS), double negative (DNG), and single negative (SNG)]. Dispersion curve analysis was used to study surface wave propagation for DPS, DNG, and SNG configurations for the TE and TM modes, and it was reported that the SNG configuration does not support surface wave propagation while the DNG configuration supports surface wave propagation for both modes^19,20^. After that, a finite difference time domain (FDTD)-based numerical study was carried out to probe the existence of surface waves at the interface of DNG–SNG media. Three different unit cells for split-ring resonators (SRRs) were used, and the existence of surface waves along the DNG–SNG interface was reported. The dispersion curves for the first Brillouin zone were presented, and the localization of surface waves on the edge was reported^21,22^.

In parallel, Ruppin studied the propagation of surface polaritons on the interface of a free space-left-handed material slab in the microwave frequency range and deduced degenerate modes for both the TE and TM modes^23^. Moreover, to physically excite the surface polaritons on left-handed metamaterials, the attenuated reflection technique (ART) was also implemented. The conversion of propagating waves to surface waves is still a big challenge for the optics research community, because the direct excitation of surface waves is not possible due to the momentum mismatch. However, a recent study revealed that the graded index metasurface can be used as a compensator that can efficiently transform a propagating wave into a surface wave with almost 100% efficiency in the microwave region^24^. In contrast, Svetlana et al. proposed an exact Eigen mode solution for a periodical impenetrable metasurface structure that can be used for the conversion of surface waves into propagating waves for the design of high-efficiency leaky wave antennae^25^.

La Spada et al. studied a new scheme for the manipulation and control of surface waves by using a curvilinear metasurface. This study revealed that the curvature plays an important role in the control of the phase and amplitude of surface waves and their cloaking applications in microwave regimes^26^. Keeping in mind these tremendous applications and the extraordinary degree of freedom to manipulate the electromagnetic energy by metasurfaces, the present study examines a resistive metasurface-covered metamaterial grounded slab. The surface wave propagation along the interface of the metasurface and metamaterial is modeled for TE and TM polarization. The surface wave propagation for two configurations for metamaterials (i.e., DPS and DNG) is discussed in the present study. In “Formulations and methodology” section discusses the electromagnetic field phasors of surface waves for the TM and TE polarized modes and their corresponding boundary conditions, while the numerical modeling of the resistive metasurface and metamaterial along with their associated results and a discussion dealing with the dispersion curves, effective mode index, field profiles, and phase speed of surface waves is presented in “Results and discussion” section. Section 4 draws several conclusions.

## Formulations and methodology

**Figure 1:**
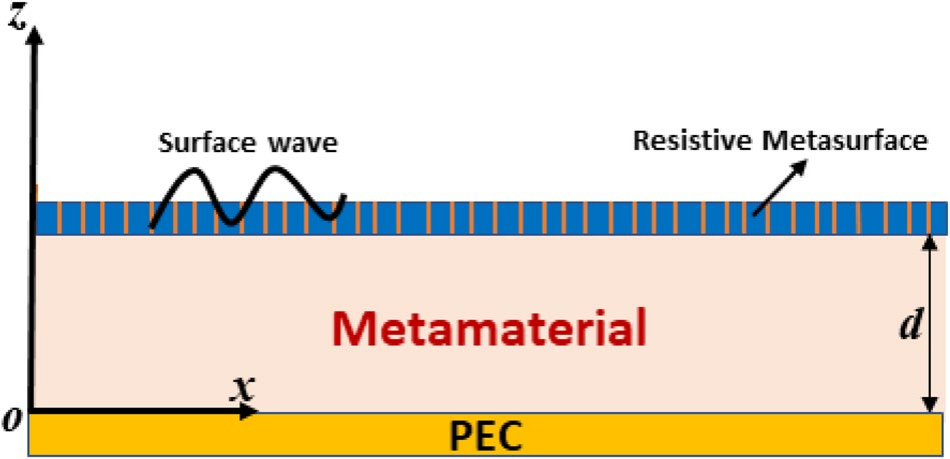
Geometry of resistive metasurface-covered grounded metamaterial for surface wave propagation.

In this section, the analytical modeling of the field phasors of electromagnetic surface waves supported by the resistive metasurface-covered grounded metamaterial structure is presented. The electromagnetic surface waveguide structure comprises three different layers of materials that are modeled as a function of space along the z-axis. The region $0 \le z \le$ d is taken as a metamaterial slab of thickness ($d$) with constitutive parameters $\left( {\varepsilon_{1} \left( \omega \right),\mu_{1} \left( \omega \right)} \right)$ and backed by the perfect electric conductor (PEC), while the metasurface is modeled as a very thin resistive sheet of thickness ($t$), with surface resistance $\left( {R_{s} } \right)$ and effective permittivity ($\varepsilon_{r} )$, as presented in Fig. 1.

The electromagnetic surface waves highly depend upon the mode of polarization (i.e., TM or TE mode). Ordinary material only supports the TM polarized wave mode for surface wave propagation, while metamaterial supports both modes of polarization. Keeping this in mind, the analytical solution for both modes is presented in the subsequent part.

### TM polarization

The field phasors for the TM polarized mode, in which the electric field (**E**) is oriented parallel to the x-axis and the associated magnetic field (**H**) is along the y-axis, are given below for each region, as in^16,27^ for the region $z >$ d:

1 $$H_{y}^{I} = Ae^{ - i\beta x} e^{{ - k_{1} z}}$$

2 $$E_{x}^{I} = \frac{1}{{i\omega \varepsilon_{o} }}Ak_{1} e^{ - i\beta x} e^{{ - k_{1} z}}$$

where $\omega$, $\varepsilon_{o}$, and $k_{1} = \sqrt {\beta^{2} - k_{o}^{2} }$ are the frequency, permittivity of free space, and wave vector in region-I, respectively.

For the region $0 < z \le$ d,

3 $$H_{y}^{II} = Be^{ - i\beta x} \cosh \left( {k_{2} z} \right)$$

4 $$E_{x}^{II} = - \frac{1}{{i\omega \varepsilon_{1} }}Bk_{2} e^{ - i\beta x} \sinh \left( {k_{2} z} \right)$$

where $\varepsilon_{1}$, $\mu_{1}$, and $k_{2} = \sqrt {\beta^{2} - \omega^{2} \varepsilon_{1} \mu_{1} }$ are the permittivity, permeability, and wave vector for region-II, respectively, while $\beta$ is the unknown complex propagation constant for the surface wave. To model the thin resistive metasurface at the subwavelength level, the following tangential impedance boundary conditions (IBCs) are applied to the interface between the resistive metasurface and metamaterial at $z = d$^28,29^:

5 $$\left. { \begin{array}{*{20}c} {E_{x}^{I} = E_{x}^{II} } \\ 1 \\ {H_{y}^{II} - H_{y}^{II} = {\text{J}}_{{\text{S}}} } \\ \end{array} } \right\}$$

where ${\text{J}}_{{\text{s}}}$ is the surface current density and computed as ${\text{J}}_{{\text{s}}} = \frac{{E_{x}^{I} }}{{R_{s} }}$ and $R_{s}$ is the resistance of the metasurface, as given in^30^. By putting Eqs. (1) to (4) in Eq. (5), we obtain the following characteristic equation for the TM polarized mode:

6 $$\sinh \left( {k_{1} d} \right)\left( {1 + \frac{{k_{2} \eta_{o} }}{{ik_{o} R_{s} }}} \right) + k_{2} \varepsilon_{d} \cosh \left( {k_{1} d} \right) = 0$$

### TE polarization

The field phasors for the TE polarized mode, in which the electric field (**E**) is oriented parallel to the y-axis and the associated magnetic field (**H**) is along the x-axis, are given for each region, as in^16,27^, for the region $z >$ d:

7 $$E_{y}^{I} = Ae^{ - i\beta x} e^{{ - k_{1} z}}$$

8 $$H_{x}^{I} = \frac{ - A}{{i\omega \mu_{o} }}k_{1} e^{ - i\beta x} e^{{ - k_{1} z}}$$

where $\omega$, $\mu_{o}$, and $k_{1} = \sqrt {\beta^{2} - k_{o}^{2} }$ are the frequency, permeability of free space, and wave vector in region-I, respectively.

For the region $0 < z \le$ d,

9 $$E_{y}^{II} = B e^{ - i\beta x} \sinh \left( {k_{2} z} \right)$$

10 $$H_{x}^{II} = \frac{B}{{i\omega \mu_{1} }}e^{ - i\beta x} \cosh \left( {k_{2} z} \right)$$

where $\varepsilon_{1}$, $\mu_{1}$, and $k_{2}$ are the permittivity, permeability, and wave vector for region-II, respectively. To compute the unknown propagation constant $\left( \beta \right)$ for the TE polarized surface wave, the following IBCs are implemented at $z = d$^28,29^:

11 $$\left. { \begin{array}{*{20}c} {E_{y}^{I} = E_{y}^{II} } \\ 1 \\ {H_{x}^{II} - H_{x}^{I} = {\text{J}}_{{\text{S}}} } \\ \end{array} } \right\}$$

where the surface current density ${\text{J}}_{{\text{s}}}$ is given as ${\text{J}}_{{\text{s}}} = \frac{{E_{y}^{I} }}{{R_{s} }}$ and $R_{s}$ is the resistance of the metasurface. By putting the equations in Eq. (11), the characteristic equation for the TE polarized mode of the surface wave is computed as follows:

12 $$\sinh \left( {k_{1} d} \right)\left( {k_{2} + i\frac{{k_{o} \eta_{o} }}{{R_{s} }}} \right) + k_{1} \cosh \left( {k_{1} d} \right) = 0$$

## Results and discussion

In this section, the numerical simulations of the above analytically computed characteristic equations of electromagnetic surface waves for both the TM and TE polarized modes for the frequency range $\omega \in$ [1, 100] GHz are presented. All the numerical results have been computed in the Mathematica software pack. Numerical modeling of the problem has been executed in two parts i.e., modeling of resistive metasurface and modeling of metamaterial.

In the first step, the resistive metasurface has been modeled as a resistive sheet of surface resistance ($R_{s}$). Which is taken as a function of the incident frequency ($\omega )$, subwavelength thickness ($t$), and effective permittivity of the metasurface ($\varepsilon_{r} )$. The explicit analytical expression of surface resistance ($R_{s}$) for resistive metasurface is given as $R_{s} = \frac{{\eta_{o} }}{{{\text{i}}\upomega \sqrt {\varepsilon_{o} \mu_{o} } t\left( {\frac{{\varepsilon_{r} }}{{\varepsilon_{o} }} - 1} \right)}}$, as reported and numerically simulated in^31^.

Secondly, the modeling of metamaterials is done numerically for two configurations (i.e., DPS and DNG). For the case of the DPS configuration, the constitutive relations $\left( {\varepsilon_{1} ,\mu_{1} } \right)$ for region $0 < z \le$ d are simultaneously positive (i.e., $\varepsilon_{1} > 0$ and $\mu_{1} > 0$), while for the DNG configuration, simultaneously negative constitutive relations are employed (i.e., $\varepsilon_{1} < 0$ and $\mu_{1} < 0$). To simulate the realizable numerical results, the DPS metamaterial is taken as $\varepsilon_{1} = 2.2\varepsilon_{o} , 2.9\varepsilon_{o} ,3.9\varepsilon_{o} , 4.4\varepsilon_{o}$, and $\mu_{1} = \mu_{o}$, and the DNG metamaterial is taken as $\varepsilon_{1} = - \left( {2.2 + i0.01} \right)\varepsilon_{o} , - \left( {2.9 + i0.01} \right)\varepsilon_{o} , - \left( {3.9 + i0.01} \right)\varepsilon_{o} , - \left( {4.4 + i0.01} \right)\varepsilon_{o}$, and $\mu_{1} = - \mu_{o}$, as extracted from the Kramers–Kronig relations. This is based upon the causality principle reported by Stockman in^32^, and their realizations in the GHz range have been discussed by many authors in^33,34^ .

To compute the numerical solutions of characteristic Eqs. (6) and (12), the contour plotting technique is applied in the kernel to obtain the solution set of real values of β that satisfy the characteristic equations. After that, the different characteristics of surface waves (i.e., dispersion relations, field profiles, effective mode index, and phase speed) supported by the resistive metasurface-covered grounded metamaterial for both polarized modes under DPS and DNG configurations are studied. In all the results, the surface waves for the TE polarized mode for the DPS configuration are not presented, because the DPS does not support surface waves, as provided in^11^.

### Dispersion curve analysis

**Figure 2:**
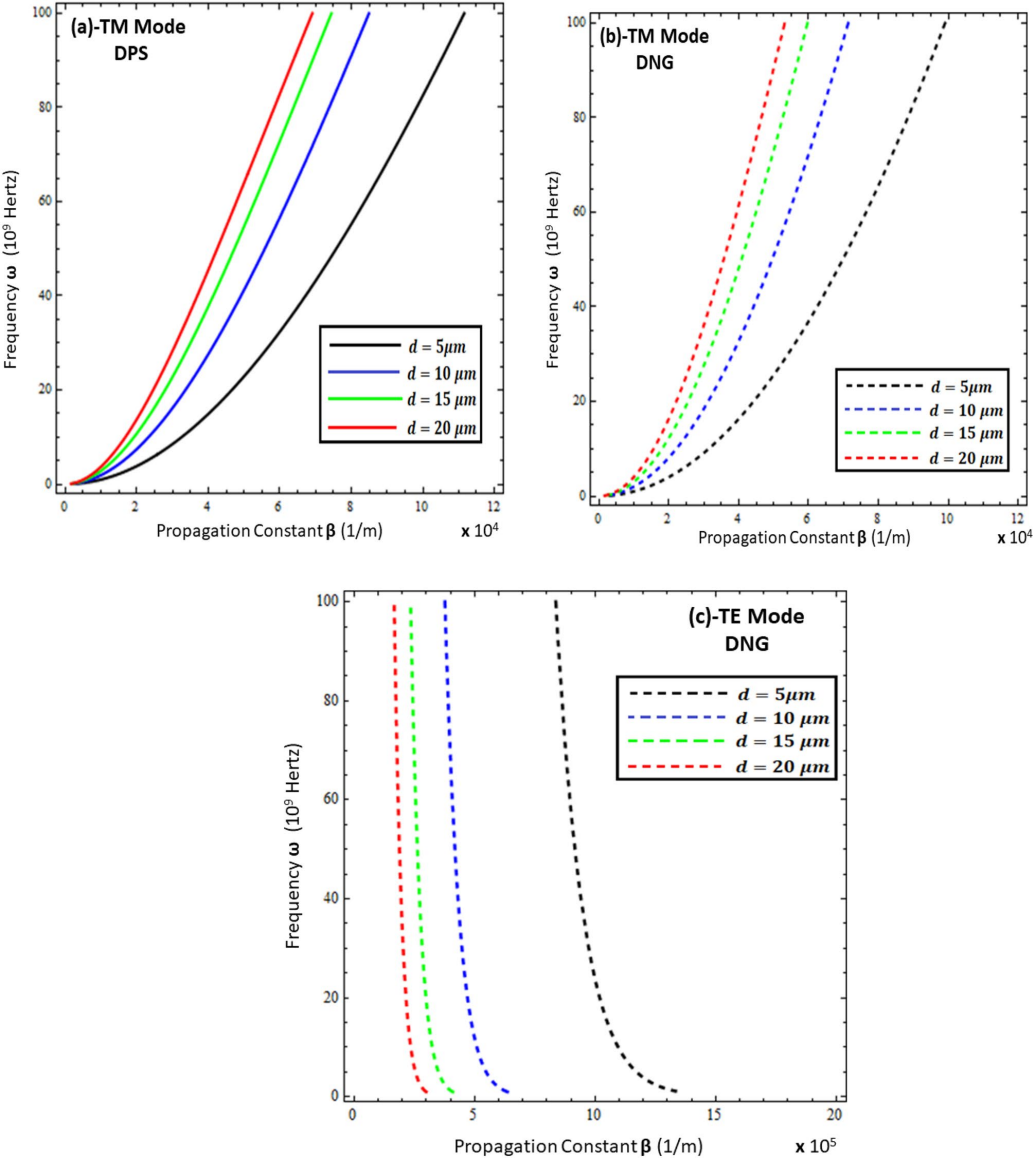
Dispersion curve analysis of surface wave under different values of metamaterial thickness. (**a**) TM polarized mode under DPS configuration with $t = 0.01 \lambda_{o}$, $\varepsilon_{r} = \left( {3.9 + i 0.1} \right)\varepsilon_{o}$, and $\varepsilon_{1} = + 4.4 \varepsilon_{o}$, (**b**) TM polarized mode under DNG configuration with $t = 0.01 \lambda_{o}$, $\varepsilon_{r} = \left( {3.9 + i 0.1} \right)\varepsilon_{o}$, and $\varepsilon_{1} = - \left( {4.4 + i 0.1} \right)\varepsilon_{o}$, (**c**) TE polarized mode under DNG configuration with $t = 0.01 \lambda_{o}$, $\varepsilon_{r} = \left( {3.9 + i 0.1} \right)\varepsilon_{o}$, and $\varepsilon_{1} = - \left( {4.4 + i 0.1} \right)\varepsilon_{o}$.

**Figure 3:**
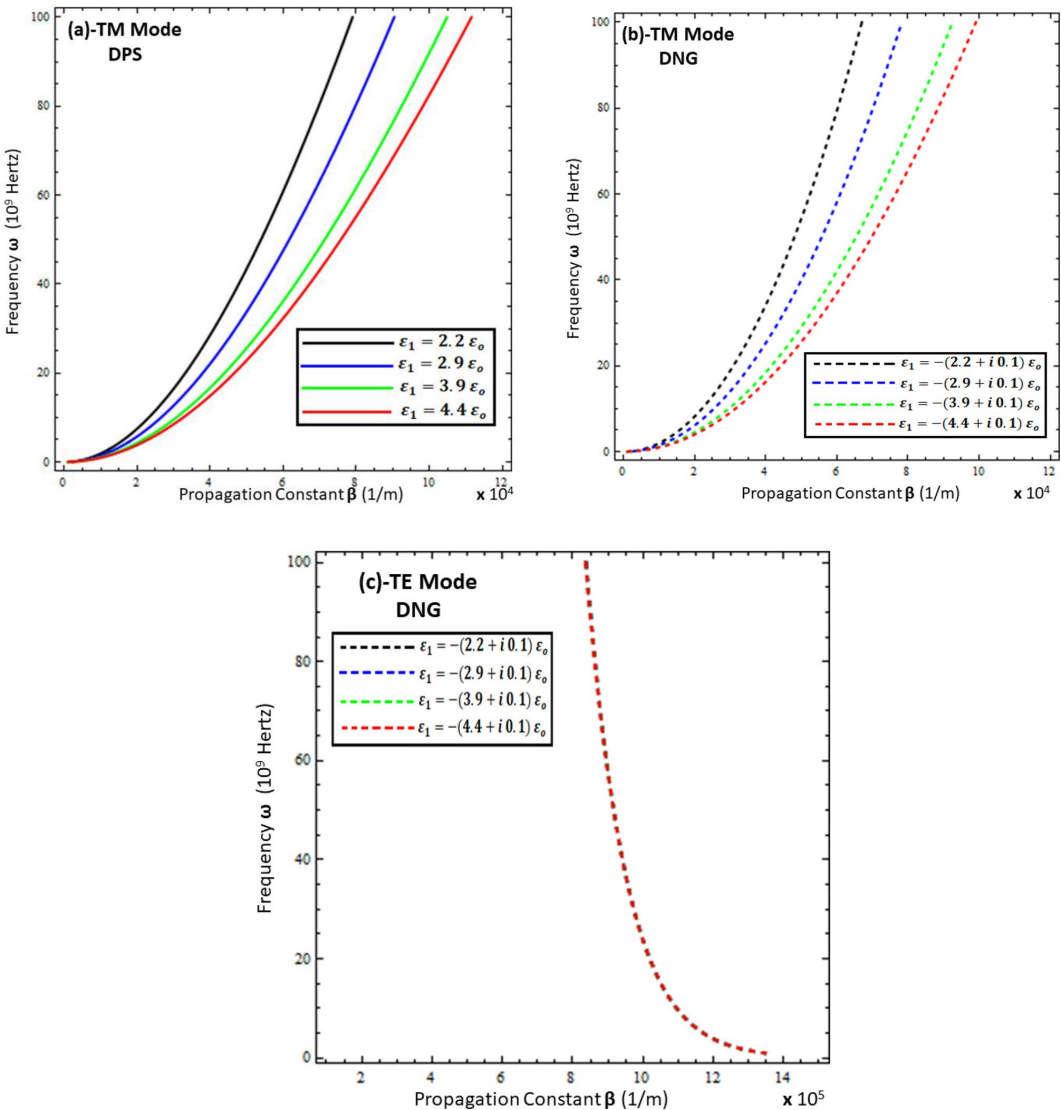
Influence of metamaterial permittivity on dispersion curves of surface wave. (**a**) TM polarized mode under DPS configuration with $t = 0.01 \lambda_{o}$, $\varepsilon_{r} = \left( {3.9 + i 0.1} \right)\varepsilon_{o}$, and *d* = 5 µm, (**b**) TM polarized mode under DNG configuration with $t = 0.01 \lambda_{o}$, $\varepsilon_{r} = \left( {3.9 + i 0.1} \right)\varepsilon_{o}$, and *d* = 5 µm, (**c**) TE polarized mode under DNG configuration with $t = 0.01 \lambda_{o}$, $\varepsilon_{r} = \left( {3.9 + i 0.1} \right)\varepsilon_{o}$, and *d* = 5 µm.

**Figure 4:**
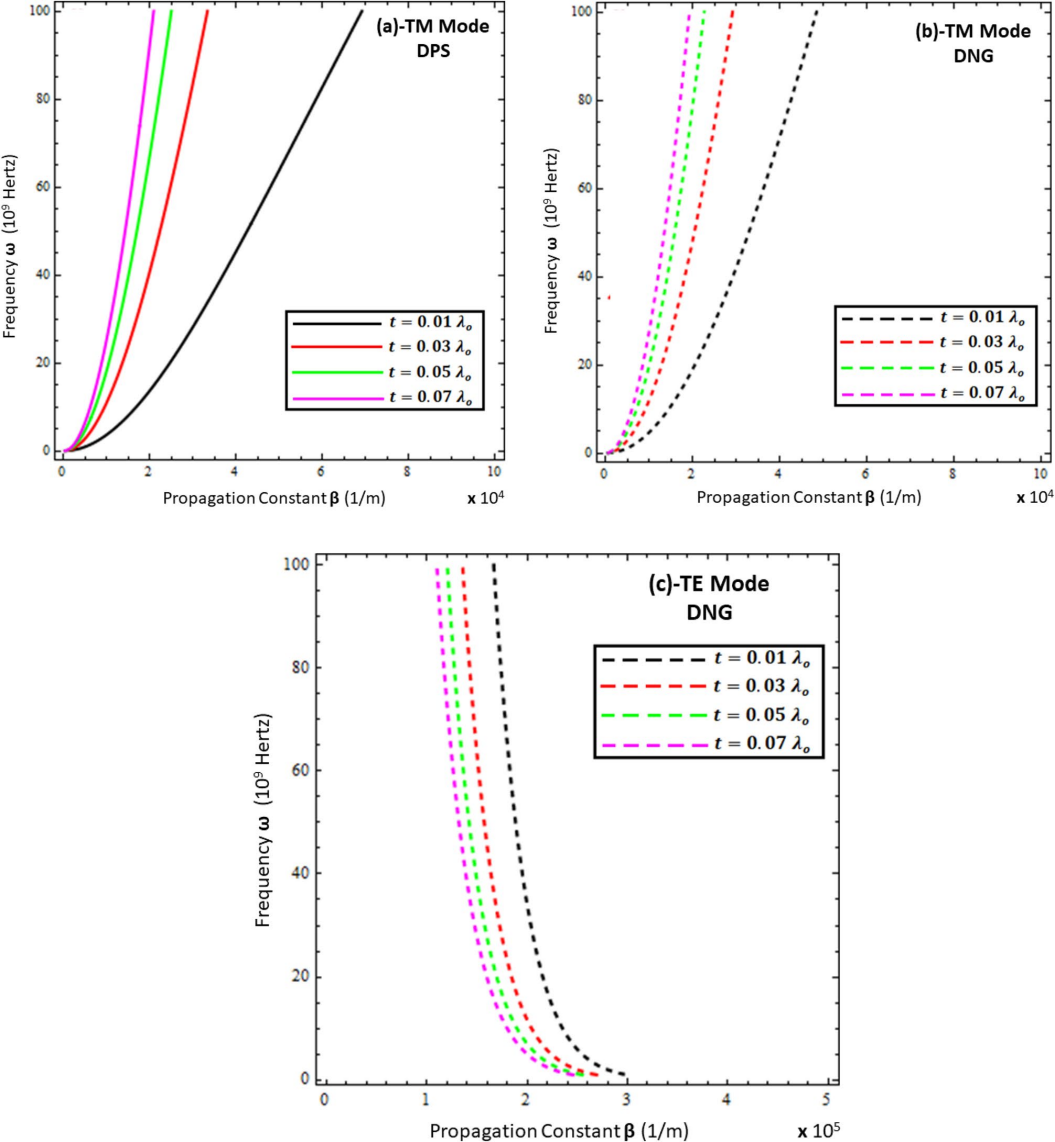
Influence of thickness of resistive metasurface on dispersion curves of surface wave. (**a**) TM polarized mode under DPS configuration with $\varepsilon_{r} = \left( {3.9 + i 0.1} \right)\varepsilon_{o}$, $\varepsilon_{1} = + 4.4 \varepsilon_{o}$, and *d* = 20 µm, (**b**) TM polarized mode under DNG configuration with $\varepsilon_{r} = \left( {3.9 + i 0.1} \right)\varepsilon_{o}$, $\varepsilon_{1} = - \left( {4.4 + i 0.1} \right)\varepsilon_{o}$, and *d* = 20 µm, (**c**) TE polarized mode under DNG configuration with $\varepsilon_{r} = \left( {3.9 + i 0.1} \right)\varepsilon_{o}$, $\varepsilon_{1} = - \left( {4.4 + i 0.1} \right)\varepsilon_{o}$ , and *d* = 20 µm.

**Figure 5:**
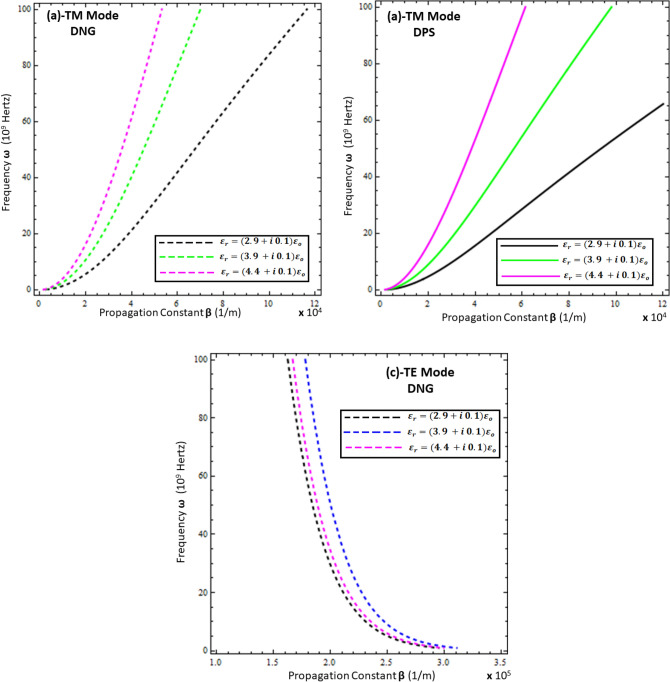
Influence of permittivity of resistive metasurface on dispersion curves of surface wave. (**a**) TM polarized mode under DPS configuration with $t = 0.01 \lambda_{o}$, $\varepsilon_{1} = + 4.4 \varepsilon_{o}$, and *d* = 20 µm, (**b**) TM polarized mode under DNG configuration with $t = 0.01 \lambda_{o}$, $\varepsilon_{1} = - \left( {4.4 + i 0.1} \right)\varepsilon_{o}$, and *d* = 20 µm, (**c**) TE polarized mode under DNG configuration with $t = 0.01 \lambda_{o}$, $\varepsilon_{1} = - \left( {4.4 + i 0.1} \right)\varepsilon_{o}$, and *d* = 20 µm.

To study the propagation characteristics of surface waves, the graphs of the relationship between the frequency $\left( \omega \right)$ and propagation constant $Re\left\{ \beta \right\}$, known as the dispersion curve, are plotted in Figs. 2, 3, 4 and 5. Figures 2 and 3 depict the influence of the thickness and permittivity of the metamaterial on the dispersion curves of surface waves for both the TM and TE polarized modes, while Figs. 4 and 5 exhibit a comparison of the dispersion curves of surface waves for the TE and TM modes under different values of thickness ($t$) and permittivity of the resistive metasurface ($\varepsilon_{r}$). It is obvious from Fig. 2a,b that the DPS and DNG metamaterial configurations support the surface wave propagation for TM polarization and that the thickness ($d$) of the metamaterial has a significant effect on the propagation constant $Re\left\{ \beta \right\}$ of surface waves for various values (i.e., *d* = 5 µm, *d* = 10 µm, *d* = 15 µm, and *d* = 20 µm). With the increase of thickness ($d$), the propagation constant decreases for both the DPS and DNG configurations. For the TE polarization case in Fig. 2c, the surface wave dispersion curve also shows quite rapid change toward the change in the thickness of the metamaterial, as discussed in^16,17^, which shows the consistency of the present work with the published work. Further, the influence of the permittivity of the DPS metamaterial for various values (i.e., $\varepsilon_{1} = + 2.2 \varepsilon_{o}$, $\varepsilon_{1} = + 2.9 \varepsilon_{o}$, $\varepsilon_{1} = + 3.9 \varepsilon_{o}$, and $\varepsilon_{1} = + 4.4 \varepsilon_{o}$) on the dispersion curve is presented in Fig. 3a for TM polarization.

To study the effect of the permittivity of the DNG metamaterial (i.e., $\varepsilon_{1} = - \left( {2.2 + i 0.1} \right) \varepsilon_{o}$, $\varepsilon_{1} = - \left( {2.9 + i 0.1} \right) \varepsilon_{o}$, $\varepsilon_{1} = - \left( {3.9 + i 0.1} \right) \varepsilon_{o}$, and $\varepsilon_{1} = - \left( {4.4 + i 0.1} \right) \varepsilon_{o}$), its influence on the dispersion curve for the TM and TE polarized modes is given in Fig. 3b,c, respectively. On the contrary, the propagation constant $Re\left\{ \beta \right\}$ starts increasing against the surface wave frequency with the increase of the permittivity ($\varepsilon_{1}$) of the metamaterial for both the DPS and DNG configurations. However, the propagation constant $Re\left\{ \beta \right\}$ remains unchanged under the influence of the metamaterial’s permittivity ($\varepsilon_{1}$) for the TE polarized DNG configuration, as depicted in Fig. 3c. The effect of the thickness of the resistive metasurface for different values (i.e., $t = 0.01 \lambda_{o}$, $t = 0.03 \lambda_{o}$, $t = 0.05 \lambda_{o}$, and $t = 0.07 \lambda_{o}$) on the dispersion curve for surface propagation on each configuration is plotted in Fig. 4, which clearly depicts that the propagation constant $Re\left\{ \beta \right\}$ can be tuned by changing the thickness of the resistive metasurface for each configuration of metamaterial for each polarization mode. The influence of the effective permittivity ($\varepsilon_{r}$) of the resistive metasurface under different values (i.e., $\varepsilon_{r} = \left( {2.9 + i 0.1} \right)\varepsilon_{o}$ , $\varepsilon_{r} = \left( {3.9 + i 0.1} \right)\varepsilon_{o}$, and $\varepsilon_{r} = \left( {4.4 + i 0.1} \right)\varepsilon_{o}$) on the dispersion curves of surface waves for each configuration is shown in Fig. 5. It is clear from Fig. 5a,b that the propagation constant $Re\left\{ \beta \right\}$ decreases with the increase of the effective permittivity of the resistive metasurface for DPS and DNG, while it shows nonlinear behavior toward the change in the effective permittivity of the resistive metasurface.

### Field profiles of surface waves

**Figure 6:**
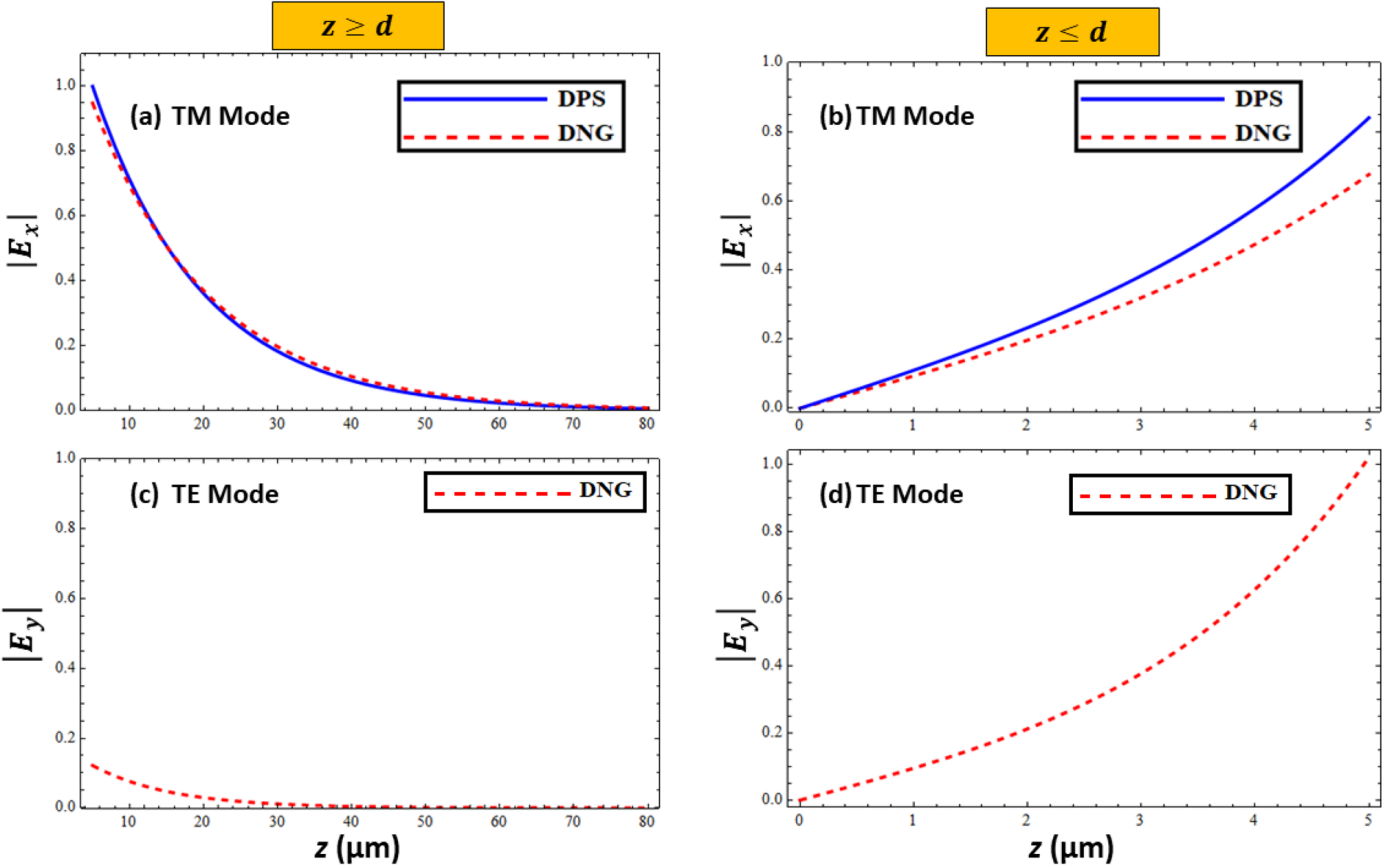
Normalized electric field profiles of surface waves as function of distance. (**a**) Comparison between TM polarized mode for DPS and DNG configurations for $z \ge d$, (**b**) for $z \le d$ with DPS configuration parameters: $\beta = 6.79 \times 10^{4} \;{\text{m}}^{ - 1}$, $\omega = 40.75 \times 10^{9} \;{\text{Hz}}$, and $\varepsilon_{1} = + 4.4\varepsilon_{o}$, for DNG configuration parameters: $\beta = 6.27 \times 10^{4} \;{\text{m}}^{ - 1}$, $\omega = 40.75 \times 10^{9} \;{\text{Hz}}$, and $\varepsilon_{1} = - \left( {4.4\varepsilon_{o} + i 0.1} \right)\varepsilon_{o}$, (**c**) TE polarized mode for DNG configuration for $z \ge d$, (**d**) for $z \le d$ with $\beta = 9.43 \times 10^{4} \;{\text{m}}^{ - 1}$, $\omega = 40.5 \times 10^{9} \;{\text{Hz}}$, and $\varepsilon_{1} = - \left( {4.4\varepsilon_{o} + i 0.1} \right)\varepsilon_{o}$, *d* = 5 µm and $\varepsilon_{r} = \left( {3.9 + i 0.1} \right)\varepsilon_{o}$.

Electromagnetic surface waves are defined as waves that propagate along the interface and decay exponentially as they move away from the interface. To verify the existence of the surface waves supported by the interface of the resistive metasurface and grounded metamaterial under different configurations and polarization modes, the normalized electric field profiles are presented in Fig. 6. Figure 6a presents a comparison of the normalized electric field strength of $\left| {{\varvec{E}}_{{\varvec{x}}} } \right|$ (TM polarized mode) for region $z \ge d$ under DPS and DNG configurations. It is obvious from Fig. 6a that $\left| {{\varvec{E}}_{{\varvec{x}}} } \right|$ decays exponentially for DPS and DNG metamaterials, as the transverse distance (z) increases from the resistive interface. A similar comparison between the normalized $\left| {{\varvec{E}}_{{\varvec{x}}} } \right|$ for DPS and DNG configurations is presented in Fig. 6b for region $z \le d$. The figure shows that $\left| {{\varvec{E}}_{{\varvec{x}}} } \right|$ decays faster for DNG as compared to DPS, as the surface wave profile moves from the resistive metasurface and metamaterial interface. It is clear that the TM polarized surface wave profile exponentially decays as it moves away from either interface (i.e., $z \ge d$ or $z \le d$), which confirms the basic property of the surface wave^11,15^. Similarly, Fig. 6c,d depict the behavior of the normalized electric field $\left| {{\varvec{E}}_{{\varvec{y}}} } \right|$ of the DNG configuration for the TE polarized mode against the transverse distance (z) for both regions $z \ge d$ and $z \le d$. It is obvious from the figures that the TE polarized surface wave mode decays sharply as it moves away from the free space in region $z \ge d$ as compared to the DNG metamaterial in region $z \le d$. This confirms that the resistive metasurface-covered DNG metamaterial for TE polarization supports surface wave propagation, which is akin to the surface polaritons discussed in^35^.

### Effective mode index of surface waves

**Figure 7:**
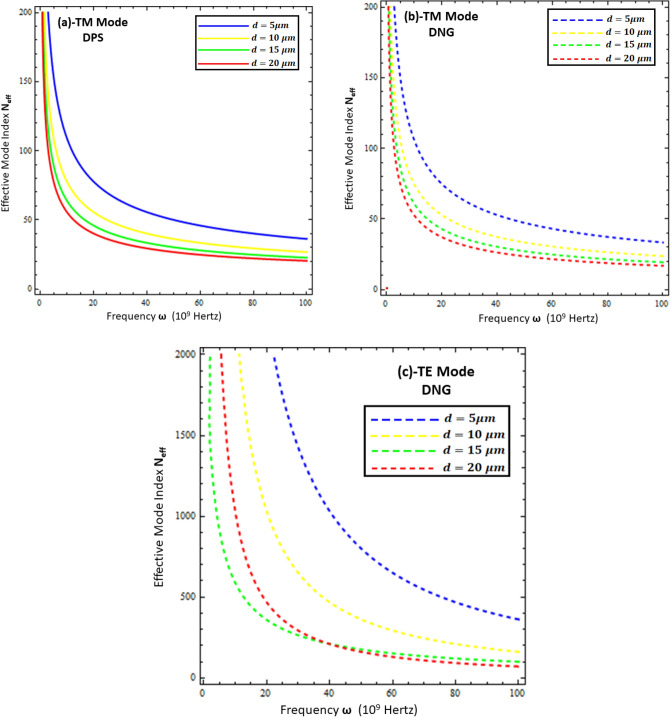
Comparison between effective mode index ($$N_{eff} )$$ under different values of metamaterial thickness. (**a**) TM polarized mode under DPS configuration with $$t = 0.02 \lambda_{o}$$, $$\varepsilon_{r} = \left( {3.9 + i 0.1} \right)\varepsilon_{o}$$, and $$\varepsilon_{1} = 4.4 \varepsilon_{o}$$, (**b**) TM polarized mode under DNG configuration with $$t = 0.02 \lambda_{o}$$, $$\varepsilon_{r} = \left( {3.9 + i 0.1} \right)\varepsilon_{o}$$, and $$\varepsilon_{1} = - \left( {4.4 + i 0.1} \right)\varepsilon_{o}$$, (**c**) TE polarized mode under DNG configuration with $$t = 0.02 \lambda_{o}$$, $$\varepsilon_{r} = \left( {3.9 + i 0.1} \right)\varepsilon_{o}$$, and $$\varepsilon_{1} = - \left( {4.4 + i 0.1} \right)\varepsilon_{o}$$.

**Figure 8:**
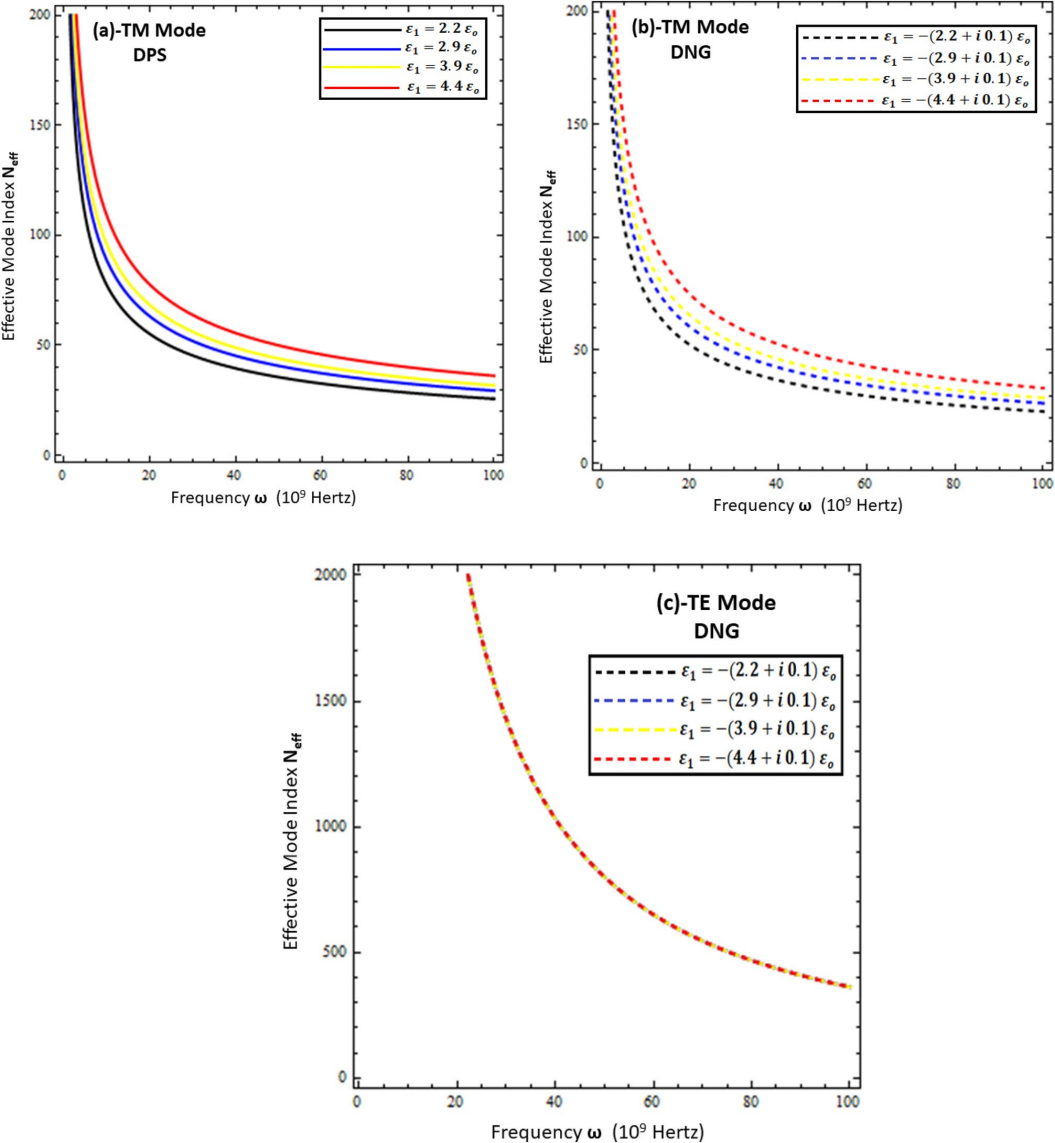
Influence of metamaterial permittivity on effective mode index ($$N_{eff} )$$. (**a**) TM polarized mode under DPS configuration with $$t = 0.02 \lambda_{o}$$, $$\varepsilon_{r} = \left( {3.9 + i 0.1} \right)\varepsilon_{o}$$, and *d* = 5 µm, (**b**) TM polarized mode under DNG configuration with $$t = 0.02 \lambda_{o}$$, $$\varepsilon_{r} = \left( {3.9 + i 0.1} \right)\varepsilon_{o}$$, and *d* = 5 µm, (**c**) TE polarized mode under DNG configuration with $$t = 0.02 \lambda_{o}$$, $$\varepsilon_{r} = \left( {3.9 + i 0.1} \right)\varepsilon_{o}$$, and *d* = 5 µm.

**Figure 9:**
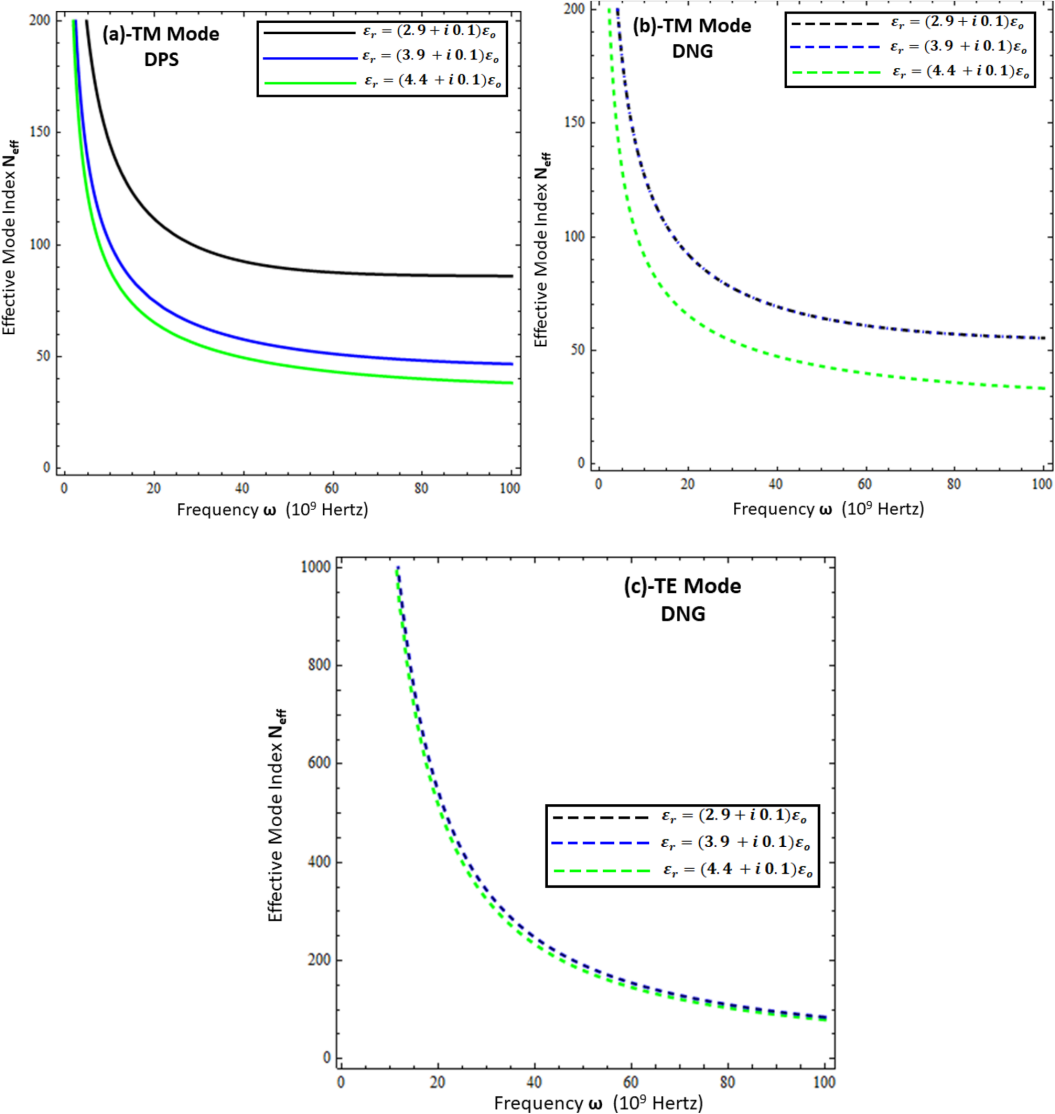
Influence of resistive permittivity on effective mode index ($$N_{eff} )$$. (**a**) TM polarized mode under DPS configuration with $$t = 0.01 \lambda_{o}$$, $$\varepsilon_{1} = + 4.4 \varepsilon_{o}$$, and *d* = 20 µm, (**b**) TM polarized mode under DNG configuration with $$t = 0.01 \lambda_{o}$$, $$\varepsilon_{1} = - \left( {4.4 + i 0.1} \right)\varepsilon_{o}$$, and *d* = 20 µm, (**c**) TE polarized mode under DNG configuration with $$t = 0.01 \lambda_{o}$$, $$\varepsilon_{1} = - \left( {4.4 + i 0.1} \right)\varepsilon_{o}$$ , and *d* = 20 µm.

**Figure 10:**
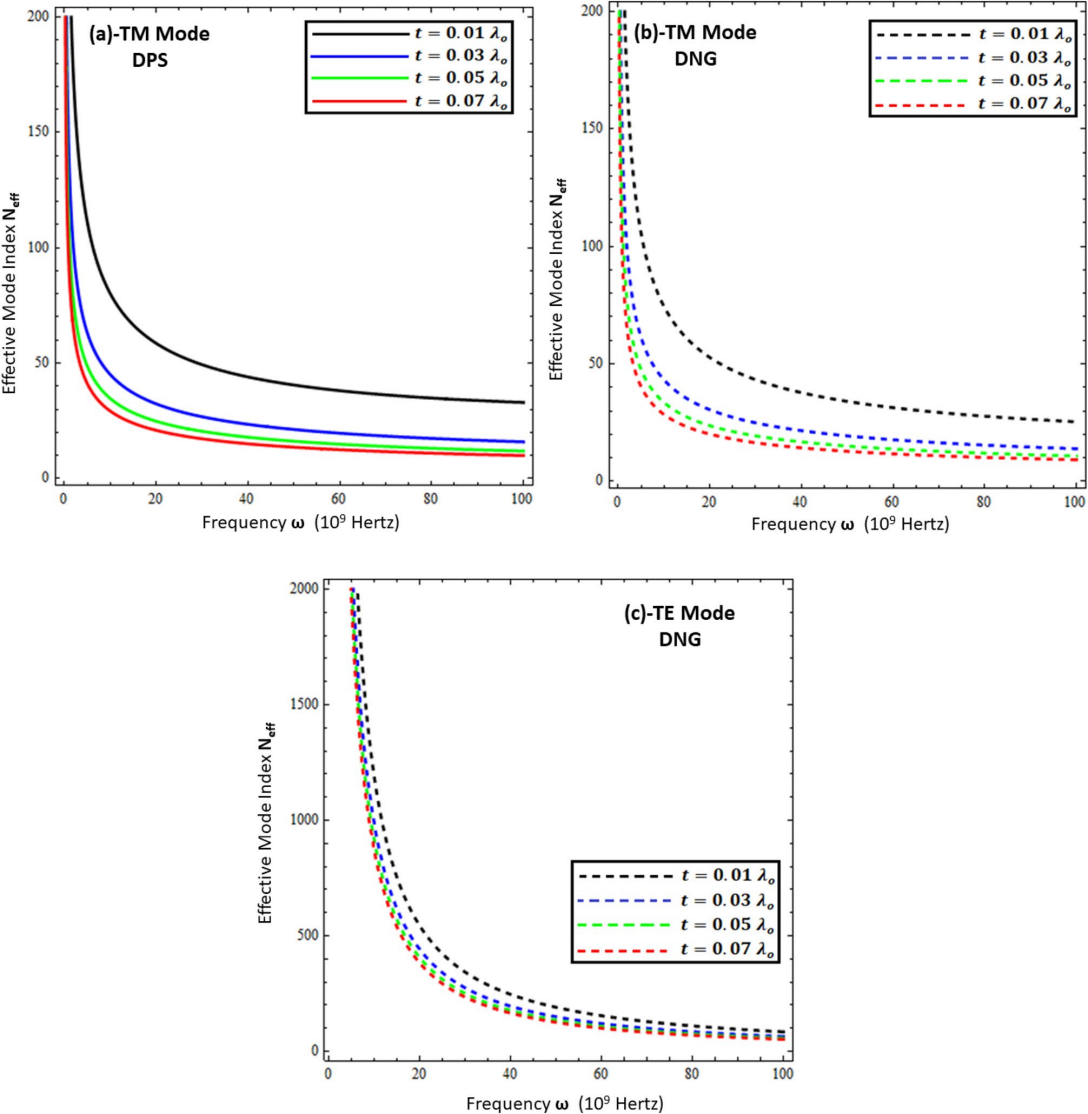
Influence of resistive sheet thickness on effective mode index ($$N_{eff} )$$. (**a**) TM polarized mode under DPS configuration with $$\varepsilon_{r} = \left( {3.9 + i 0.1} \right)\varepsilon_{o}$$, $$\varepsilon_{1} = + 4.4 \varepsilon_{o}$$, and *d* = 20 µm, (**b**) TM polarized mode under DNG configuration with $$\varepsilon_{r} = \left( {3.9 + i 0.1} \right)\varepsilon_{o}$$, $$\varepsilon_{1} = - \left( {4.4 + i 0.1} \right)\varepsilon_{o}$$, and *d* = 20 µm, (**c**) TE polarized mode under DNG configuration with $$\varepsilon_{r} = \left( {3.9 + i 0.1} \right)\varepsilon_{o}$$, $$\varepsilon_{1} = - \left( {4.4 + i 0.1} \right)\varepsilon_{o}$$, and *d* = 20 µm.

The effective mode index ($$N_{eff} )$$ is the ratio of the propagation constant surface wave $$\left( \beta \right)$$ to the free space wave number $$k_{o}$$, mathematically computed as $$N_{eff} = \frac{{Re\left\{ \beta \right\}}}{{k_{o} }}$$. Physically, it measures the confinement of the propagating wave on the interface with respect to free space propagation, and numerically, its higher values reflect the highly confined waves in the material^11,15^. To study the confinement of surface waves as a function of frequency for the present problem, each configuration is presented in Figs. 7, 8, 9 and 10. Figures 7 and 8 deal with the influence of the thickness and permittivity of DPS and DNG metamaterials for both polarized modes on the effective mode index, respectively, while Figs. 9 and 10 deal with the impact of the resistive metasurface parameters on the effective mode index for each configuration and polarization.

Figure 7 compares the effective mode index for different thicknesses of (DPS/DNG) metamaterial (i.e., *d* = 5 µm, *d* = 10 µm, *d* = 15 µm, and *d* = 20 µm), and it is obvious from the figure that the effective index of surface waves highly depends upon the thickness of the metamaterial as well as the operating frequency. The lower frequency $$\omega \in$$ [1, 20] GHz corresponds to higher effective mode index values and decays nonlinearly for each configuration, while the higher frequency $$\omega \in$$ [20, 100] GHz corresponds to lower effective mode index values. The effective mode index of surface waves for the TE mode has more values as compared to the TM mode. Figures 8a,b deal with the effective mode index as a function of frequency under different values of permittivity of the DPS metamaterial (i.e., $$\varepsilon_{1} = + 2.2 \varepsilon_{o}$$, $$\varepsilon_{1} = + 2.9 \varepsilon_{o}$$, $$\varepsilon_{1} = + 3.9 \varepsilon_{o}$$, and $$\varepsilon_{1} = + 4.4 \varepsilon_{o}$$) and DNG metamaterial (i.e., $$\varepsilon_{1} = - \left( {2.2 + i 0.1} \right) \varepsilon_{o}$$,$$\varepsilon_{1} = - \left( {2.9 + i 0.1} \right) \varepsilon_{o}$$, $$\varepsilon_{1} = - \left( {3.9 + i 0.1} \right) \varepsilon_{o}$$, and $$\varepsilon_{1} = - \left( {4.4 + i 0.1} \right) \varepsilon_{o}$$), respectively. It is clear from Fig. 8a,b that with the increase of permittivity of the DPS/DNG metamaterial, the effective mode index increases for the TM polarized surface wave mode, but for the TE polarized mode case, the effective mode index is independent of the permittivity of the metamaterial, as shown in Fig. 8c. Figures 9 and 10 deal with the influence of the thickness ($$t$$) and permittivity $$\left( {\varepsilon_{r} } \right)$$ of the resistive metasurface on the effective mode index. The effective mode index ($$N_{eff} )$$ as a function of frequency ($$\omega )$$ is studied against different values of the permittivity of the resistive metasurface (i.e., $$\varepsilon_{r} = \left( {2.9 + i 0.1} \right)\varepsilon_{o}$$ , $$\varepsilon_{r} = \left( {3.9 + i 0.1} \right)\varepsilon_{o}$$, and $$\varepsilon_{r} = \left( {4.4 + i 0.1} \right)\varepsilon_{o}$$) in Fig. 9, and it is clear that with the increase of resistive permittivity, the effective mode index decreases for the TM polarized surface wave for DPS and DNG configurations, but the influence on the TE polarized surface wave for the DNG configuration is negligible. To study the dependence of the geometrical parameters of the resistive metasurface on the confinement of the surface wave, the effective mode index $$\left( {N_{eff} } \right)$$ is plotted against frequency ($$\omega )$$ under different values of the thickness of the resistive metasurface (i.e., $$t = 0.01 \lambda_{o}$$ , $$t = 0.03 \lambda_{o}$$, $$t = 0.05 \lambda_{o}$$, and $$t = 0.07 \lambda_{o}$$) in Fig. 10. It shows that confinement of the surface wave highly depends upon the thickness of the resistive metasurface: with the increase of thickness, the effective mode index decreases for each configuration and polarization.

### Phase speed of surface waves

**Figure 11:**
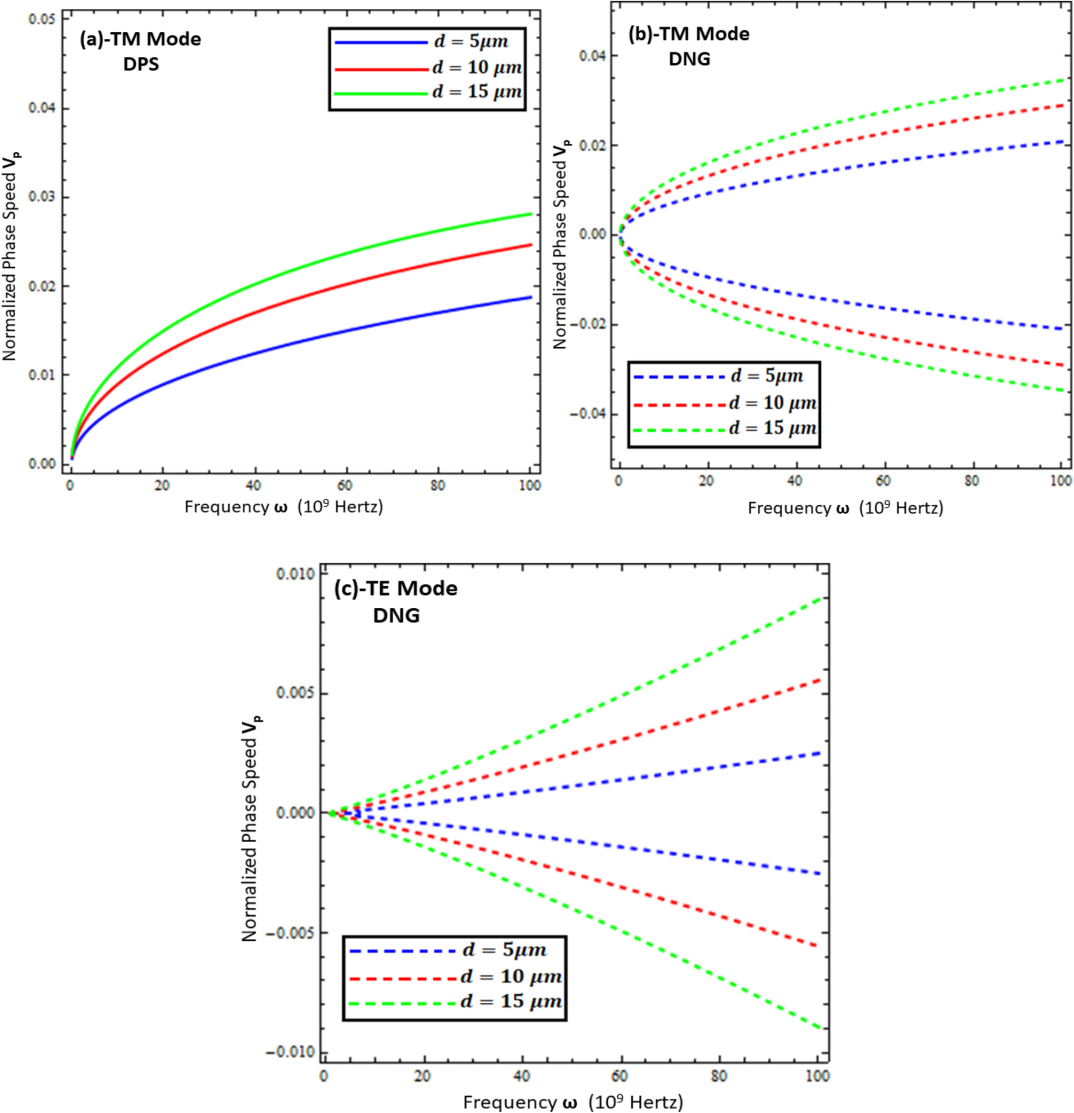
Normalized phase speed ($$V_{p} )$$ under different values of metamaterial thickness. (**a**) TM polarized mode for DPS configuration with $$t = 0.01 \lambda_{o}$$, $$\varepsilon_{r} = \left( {3.9 + i 0.1} \right)\varepsilon_{o}$$, and $$\varepsilon_{1} = + 4.4 \varepsilon_{o}$$, (**b**) TM polarized mode for DNG configuration with $$t = 0.01 \lambda_{o}$$, $$\varepsilon_{r} = \left( {3.9 + i 0.1} \right)\varepsilon_{o}$$, and $$\varepsilon_{1} = - \left( {4.4 + i 0.1} \right)\varepsilon_{o}$$, (**c**) TE polarized mode for DNG configuration with $$t = 0.01 \lambda_{o}$$, $$\varepsilon_{r} = \left( {3.9 + i 0.1} \right)\varepsilon_{o}$$, and $$\varepsilon_{1} = - \left( {4.4 + i 0.1} \right)\varepsilon_{o}$$.

**Figure 12:**
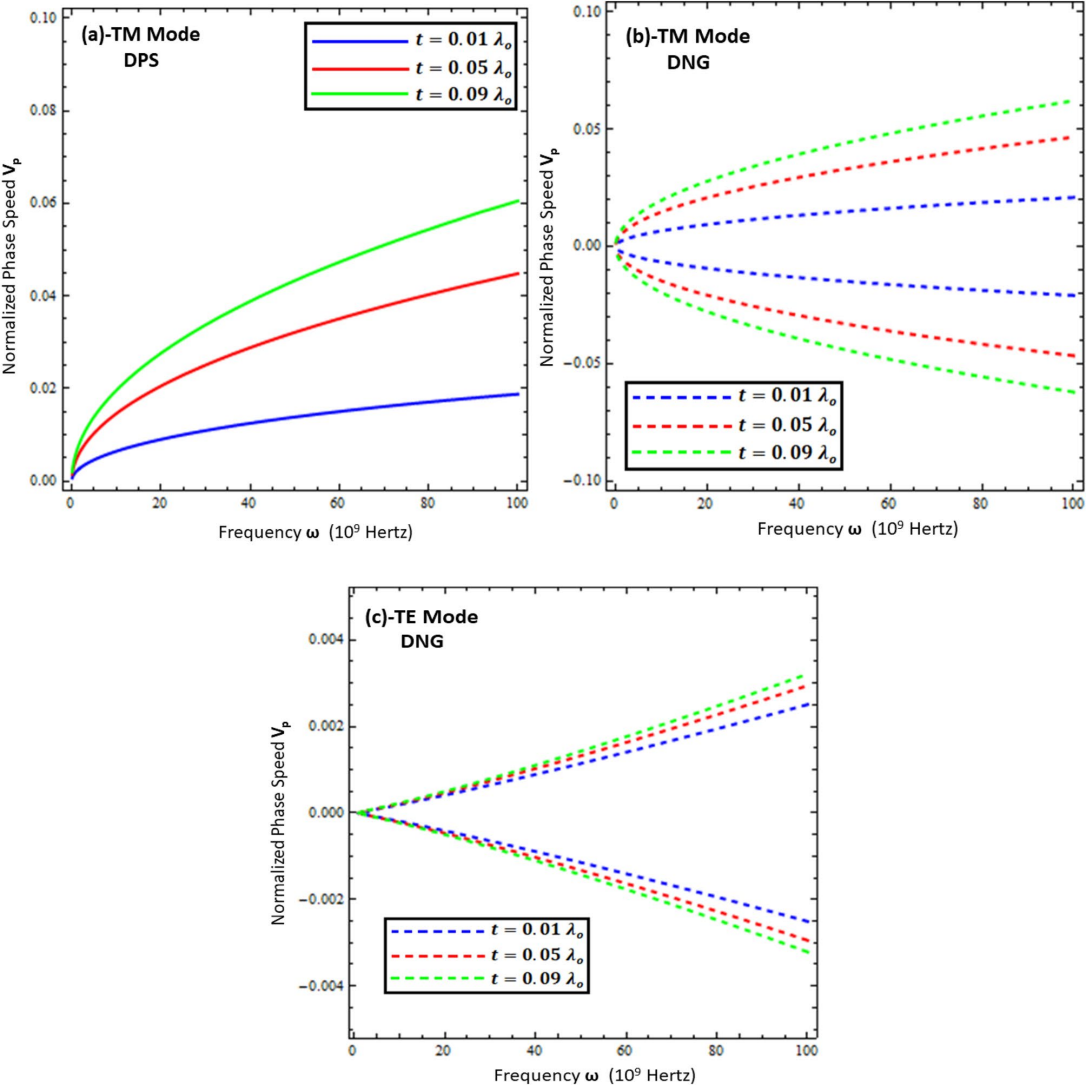
Normalized phase speed ($$V_{p} )$$ under different values of thickness of resistive metasurface. (**a**) TM polarized mode for DPS configuration with *d* = 5 µm , $$\varepsilon_{r} = \left( {3.9 + i 0.1} \right)\varepsilon_{o}$$, and $$\varepsilon_{1} = + 4.4 \varepsilon_{o}$$, (**b**) TM polarized mode for DNG configuration with *d* = 5 µm, $$\varepsilon_{r} = \left( {3.9 + i 0.1} \right)\varepsilon_{o}$$, and $$\varepsilon_{1} = - \left( {4.4 + i 0.1} \right)\varepsilon_{o}$$, (**c**) TE polarized mode for DNG configuration with *d* = 5 µm, $$\varepsilon_{r} = \left( {3.9 + i 0.1} \right)\varepsilon_{o}$$, and $$\varepsilon_{1} = - \left( {4.4 + i 0.1} \right)\varepsilon_{o}$$.

From Figs. 7, 8, 9 and 10, it can be concluded that most of the dynamics of surface waves can be controlled mainly geometrically by varying the thickness of the metamaterial and resistive metasurface. Keeping this in mind, the normalized phase speed ($v_{p}$) is studied for the TM and TE polarized surface waves for DPS and DNG configurations, as given in Figs. 11 and 12. The phase speed is normalized by the speed of light in free space ($c$) and computed as $v_{p} = \frac{1}{c}\frac{{k_{o} }}{{Re\left\{ \beta \right\}}}$ in the present work. It is clear from Figs. 11 and 12 that the phase speed of the surface waves for both configurations can be controlled by just changing the geometry of the structure (i.e., thickness of the metamaterial ($d$) and thickness of resistive metasurface ($t$). The fractional values of the phase speed of surface waves indicate the speed reduction order. Figure 11 depicts the phase speed reduction of surface waves for the TM and TE modes against the frequency under different values of metamaterial thickness (i.e., *d* = 5 µm, *d* = 10 µm, and *d* = 15 µm). It is obvious from Fig. 11a,b that the phase speed increases nonlinearly with the increase of thickness for the TM polarized DPS and DNG metamaterial configurations, respectively, while for the case of the TE polarized DNG configuration, as given in Fig. 11c, the phase speed increases monotonically with the increase of thickness ($d$). Similar trends for the phase speed of surface waves are presented in Fig. 12 when the resistive metasurface thickness is taken as $t = 0.01 \lambda_{o}$, $t = 0.05 \lambda_{o}$, and $t = 0.09 \lambda_{o}$. On comparing, it is clear that the TE mode of surface wave supported by the DNG structures provides ultraslow surface waves as compared to the other configurations of surface waves. The DNG metamaterial configuration supports the backward surface wave propagation, as discussed in^36^. Due to this reason, the negative phase velocity is accounted for in Figs. 11b,c and 12b,c. Ultraslow surface wave propagation may be used for the reduction of propagation losses, optical switching, sensing, and modulation applications, as discussed in^37,38^.

## Conclusion

The propagation of electromagnetic surface waves from the resistive metasurface grounded metamaterial structure has been studied for the TM and TE polarized modes under DPS/DNG configurations of metamaterial. From the numerical results computed in the previous section, the following conclusions can be drawn:

1. The surface waves propagate along the interface of resistive metasurface-covered DPS and DNG metamaterial configurations for the TM polarized mode only, while the TE polarized surface wave propagates only for the DNG configuration.
2. The dispersion curves of surface waves are found to be sensitive to the thickness and effective permittivity of the metamaterial as well as the resistive metasurface.
3. The confinement of the surface waves highly depends on the polarization mode: TE polarized surface waves have a higher effective mode index as compared to TM polarized surface waves.
4. The effective mode index ($N_{eff}$) was found to be sensitive to the geometrical parameters of the proposed surface waveguide structure.
5. The field profiles decay exponentially as the transverse distance increases from either interface, which confirms the basic property of surface waves.
6. The phase speed of surface waves can be controlled by varying the geometrical parameters [i.e., thickness of metamaterial ($d$) and thickness of resistive metasurface ($t$)]. Moreover, the reduction factor is much higher for TE polarized surface waves as compared to TM polarized surface waves.
7. The present work may have potential applications in surface communication, HF surface waveguide design, surface wave speed controllers, and light trapping configurations.

## Supplementary information

Supplementary file1
