# Supplementary material for: Electromagnetic surface waves supported by a resistive metasurface-covered metamaterial structure
Source: Sci Rep. 2020 Sep 23;10:15548. doi: 10.1038/s41598-020-72396-7 (PMC7511986; doi:10.1038/s41598-020-72396-7)
Supplement: Supplementary file 1 — Supplementary file1 [file 41598_2020_72396_MOESM1_ESM.docx]

Supplementary Information for

**Electromagnetic Surface Waves Supported by a Resistive Metasurface-covered Metamaterial Structure**

M. Z. Yaqoob^1,2^, A. Ghaffar^1^, Majeed A.S. Alkanhal^3^, M.Y.Naz^1^ , Ali H. Alqahtani,^4^ and Y Khan^3^

^1^ Department of Physics, University of Agriculture, Faisalabad, Pakistan.

^2^ Department of Physics, Government College University, Faisalabad, Pakistan.

^3^Department of Electrical Engineering, King Saud University, Saudi Arabia.

^4^Department of Electrical Engineering, College of Applied Engineering, King Saud University, Al-Muzahimiyah Branch, Saudi Arabia

^1^zeeshaan32@yahoo.com, ^2^aghaffar16@uaf.edu.pk, ^3^majeed@ ksu.edu.sa, [^4^yasin603@yahoo.com](mailto:4yasin603@yahoo.com), [^5^ahqahtani@ksu.edu.sa](mailto:5ahqahtani@ksu.edu.sa), ^6^yasink@ ksu.edu.sa

Corresponding Author email address: majeed@ ksu.edu.sa, [aghaffar16@uaf.edu.pk](mailto:aghaffar16@uaf.edu.pk),

**Materials and Method:**

To compute the analytical solution for the electromagnetic surface waves supported by the resistive metasurface-covered grounded metamaterial structure, a step by step solution has been presented in this section. The modeling of the problem comprises of three major parts i.e., analytical solution for propagating electromagnetic surface wave, modeling of resistive metasurface and numerical simulation as depicted in the following flow chart.


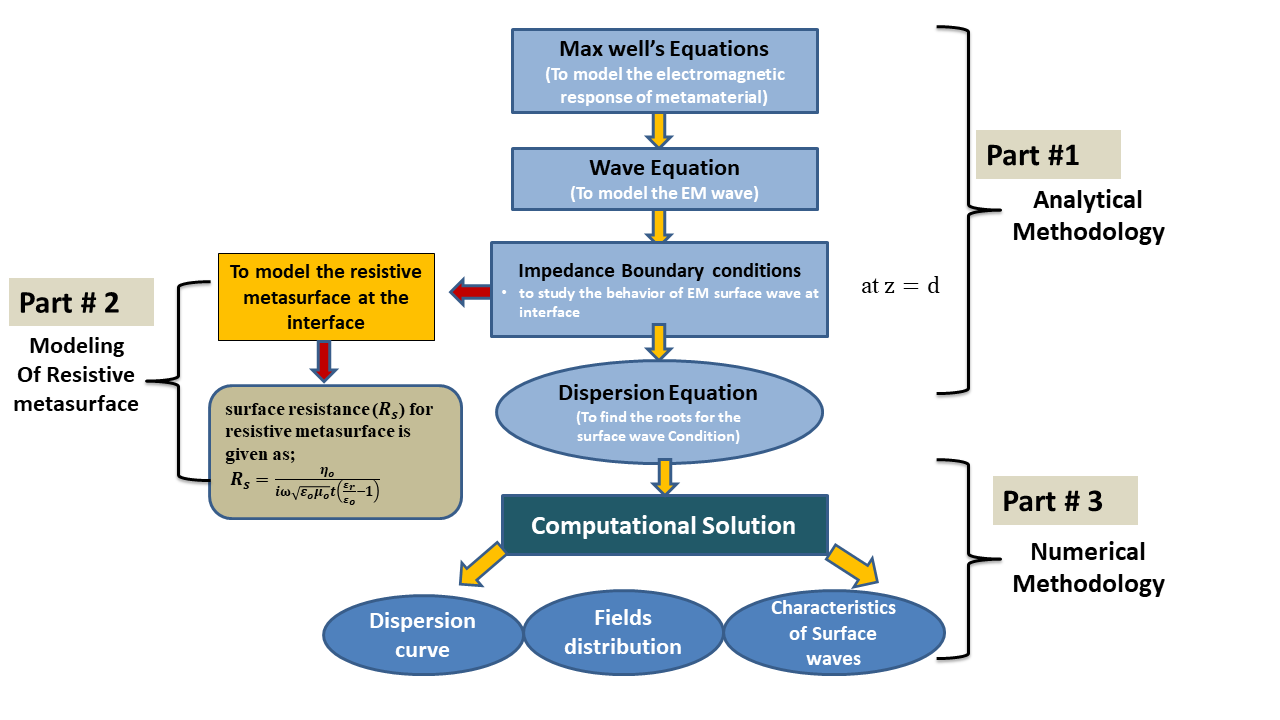


Fig. 1. Flow chart of mathematical modeling of the problem

To characterized the space, the permittivity $\varepsilon\left( z \right)$ is taken as the function of z-axis. The region i.e., $0\leq z<d$, comprised of source free isotropic, homogenous and linear metamaterial backed by the perfect electric conductor (PEC) while the region $z>d$ is taken as free space, as depicted in the geometry of the problem. First of all, the Maxwell’s equations have been solved to find out the solution of wave equation according to each region of space. The electric field $\mathbf{E}\boldsymbol{(}x,y,z,t)$ and magnetic field $\mathbf{H}\boldsymbol{(}x,y,z,t)$ is supposed to be continuous with respect of scape and time. The ${\hat{\boldsymbol{u}}}_{\boldsymbol{x}}$, ${\hat{\boldsymbol{u}}}_{\boldsymbol{y}}$, & ${\hat{\boldsymbol{u}}}_{\boldsymbol{z}}$ are the unit vectors along x, y & z-axis respectively. The dependence of fields on time is taken as $e^{+i\omega t}$. According to the source free curl Maxwell’s Equations for free space region,

$$\boldsymbol{\nabla}\times\boldsymbol{E}=-\mu\frac{\partial\boldsymbol{H}}{\partial t}\ldots\ldots\ldots..\ldots\ldots\left( 1 \right)$$

$$\nabla\times\boldsymbol{H}=\frac{\partial\boldsymbol{D}}{\partial t} \ldots\ldots\ldots\ldots..\ldots\ldots.\left( 2 \right)$$

For the time harmonic fields, time derivative $\frac{\partial}{\partial t}$ is replaced by the factor $+i\omega$. By using the curl operation, eq. (1) becomes

$$\boldsymbol{\nabla}\times\boldsymbol{E}=\left| \begin{matrix} \hat{u}_{x} & \hat{u}_{y} & \hat{u}_{z} \\ \frac{\partial}{\partial x} & \frac{\partial}{\partial y} & \frac{\partial}{\partial z} \\ E_{x} & E_{y} & E_{z} \end{matrix} \right|$$

$$\frac{\partial E_{z}}{\partial y}-\frac{\partial E_{y}}{\partial z}=-i\omega\mu H_{x}\ldots\ldots\ldots\ldots.\left( 1a \right),$$

$$\frac{\partial E_{z}}{\partial x}-\frac{\partial E_{x}}{\partial z}=i\omega\mu H_{y}\ldots\ldots\ldots\ldots.\left( 1b \right),$$

$$\frac{\partial E_{y}}{\partial x}-\frac{\partial E_{x}}{\partial y}=-i\omega\mu H_{z}\ldots\ldots\ldots\ldots.\left( 1c \right).$$

Similarly, same for the equation (2),

$$\frac{\partial H_{z}}{\partial y}-\frac{\partial H_{y}}{\partial z}=i\omega\varepsilon E_{x}\ldots\ldots\ldots\ldots.\left( 2a \right),$$

$$\frac{\partial H_{z}}{\partial x}-\frac{\partial H_{x}}{\partial z}=i\omega\varepsilon E_{y}\ldots\ldots\ldots\ldots.\left( 2b \right),$$

$$\frac{\partial H_{y}}{\partial x}-\frac{\partial H_{x}}{\partial y}=i\omega\varepsilon E_{z}\ldots\ldots\ldots\ldots.\left( 2c \right),$$

To compute the solution of surface wave equation, the above coupled equations should be uncoupled by applying the respective partial derivatives. In the present study, the propagation of electromagnetic surface wave is being considered only in the x-direction with complex valued propagating constant $(\beta)$. Moreover, the fields have no spatial variation in the perpendicular, in-plane y-direction. Mathematically, the electric field for the surface wave can be prescribed as $\mathbf{E}\left( x,y,z \right)=\mathbf{E}\left( z \right)e^{i\beta x}$ with the field variations with respect to x and y can be modeled as $\frac{\partial}{\partial x}=i\beta$ and $\frac{\partial}{\partial y}=0$.


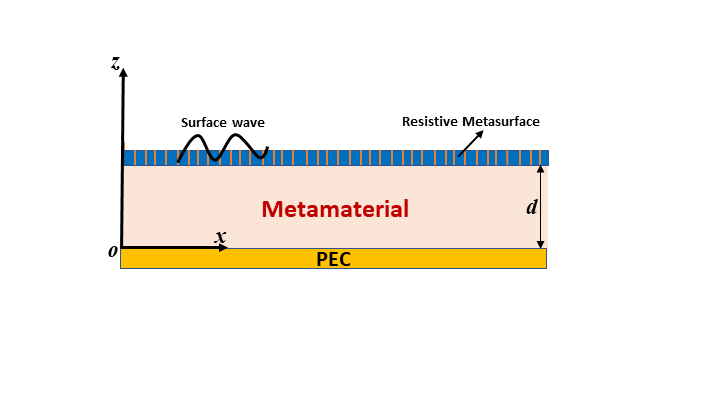


Fig.2. Geometry of resistive metasurface-covered grounded metamaterial for surface wave propagation.

By implementing these conditions, the equations 1(a)-1(c) and 2(a)-2(c) can be simplified as

$$\frac{\partial E_{y}}{\partial z}=-i\omega\mu H_{x}\ldots\ldots\ldots\ldots.\left( 3a \right),$$

$$i\beta E_{z}-\frac{\partial E_{x}}{\partial z}=i\omega\mu H_{y}\ldots\ldots\left( 3b \right),$$

$$\beta E_{y}=-\omega\mu H_{z}\ldots\ldots\ldots\ldots.\left( 3c \right).$$

Similarly, for the equation (2), on expanding the curl equation with respect

$$\frac{\partial H_{y}}{\partial z}=i\omega\varepsilon E_{x}\ldots\ldots\ldots\ldots\ldots\ldots.\left( 4a \right),$$

$$i\beta H_{z}-\frac{\partial H_{x}}{\partial z}=i\omega\varepsilon E_{y}\ldots\ldots\ldots\ldots\left( 4b \right),$$

$$\beta H_{y}=\omega\varepsilon E_{z}\ldots\ldots\ldots\ldots\ldots\ldots.\left( 4c \right),$$

The above set of six equations can be extracted as two independent polarized surface wave modes i.e., transverse magnetic (TM) and transverse electric (TE). For the TM polarized surface wave modes, the field equation reduces to

$$E_{x}=\frac{1}{i\omega\varepsilon}\frac{\partial H_{y}}{\partial z}\ldots\ldots\ldots\ldots.\left( 5a \right)$$

$$E_{z}=\frac{\beta}{\omega\varepsilon}H_{y}\ldots\ldots\ldots.\left( 5b \right),$$

and wave equation for TM polarized mode is governed as

$$\frac{\partial^{2}H_{y}}{{\partial z}^{2}}+\left( {k_{o}}^{2}\varepsilon-\beta^{2} \right)H_{y}=0\ldots\ldots\ldots\ldots\left( 5c \right)$$

where the $k_{o}$, $\varepsilon$ and $\beta$ represents the wavevector in free space, relative permittivity of the material and unknown propagation constant respectively. For the TE modes, the governing equations reduce to

$$H_{x}=\frac{-1}{i\omega\varepsilon}\frac{\partial E_{y}}{\partial z}\ldots\ldots\ldots\ldots.\left( 6a \right)$$

$$H_{z}=-\frac{\beta}{\omega\varepsilon}E_{y}\ldots\ldots\ldots.\left( 6b \right),$$

and wave equation for TE polarized mode is given as

$$\frac{\partial^{2}E_{y}}{{\partial z}^{2}}+\left( {k_{o}}^{2}\varepsilon-\beta^{2} \right)E_{y}=0\ldots\ldots\ldots\ldots\left( 6c \right)$$

The above set of partial differential equations can be solved by applying the separable variable technique and computed the solution for each region of space. On computing the general solution for the TM Polarized surface waves, for the region of free space i.e., $z>d$, the $e^{-k_{1}z}$ is obtained for the +z-axis. The solution represents the evanescent wave, which decay as the distance along z-axis increase and the $k_{1}=\sqrt{\beta^{2}-{k_{o}}^{2}}$ is the wavevector of surface wave in free space. For the solution of surface waves in the metamaterial baked by the PEC region i.e., $0\leq z<d$, the solution obtained in terms $\sinh\left( k_{2}z \right)$ and $\cosh\left( k_{2}z \right)$ as resultant of superposition of the evanescent waves in bounded region. while the $k_{2}=\sqrt{\beta^{2}-\omega^{2}\varepsilon_{1}\mu_{1}}$ is the wavevector in metamaterial with the permittivity ($\varepsilon_{1}$) and permeability ($\mu_{1}$) respectively. Similarly, the analytical solution has been obtained for the TE polarized surface waves. after this, to simulate the resistive metasurface on the interface between the free space and metamaterial the impedance boundary conditions have been employed at z=d and dispersion equations have been computed for both polarized surface waves.
